# Bioinformatics approach to predict target genes for dysregulated microRNAs in hepatocellular carcinoma: study on a chemically-induced HCC mouse model

**DOI:** 10.1186/s12859-015-0836-1

**Published:** 2015-12-10

**Authors:** Filippo Del Vecchio, Francesco Gallo, Antinisca Di Marco, Valentina Mastroiaco, Pasquale Caianiello, Francesca Zazzeroni, Edoardo Alesse, Alessandra Tessitore

**Affiliations:** Department of Biotechnological and Applied Clinical Sciences, University of L’Aquila, Via Vetoio, Coppito 2, 67100 L’Aquila, Italy; Department of Computer Engineering and Science, and Mathematics, University of L’Aquila, Via Vetoio, Coppito 1, L’Aquila, 67100 Italy

**Keywords:** MicroRNA, hepatocellular carcinoma, HCC mouse model, diethylnitrosamine, DEN, target prediction

## Abstract

**Background:**

Hepatocellular carcinoma (HCC) is an aggressive epithelial tumor which shows very poor prognosis and high rate of recurrence, representing an urgent problem for public healthcare. MicroRNAs (miRNAs/miRs) are a class of small, non-coding RNAs that attract great attention because of their role in regulation of processes such as cellular growth, proliferation, apoptosis. Because of the thousands of potential interactions between a single miR and target mRNAs, bioinformatics prediction tools are very useful to facilitate the task for individuating and selecting putative target genes. In this study, we present a chemically-induced HCC mouse model to identify differential expression of miRNAs during the progression of the hepatic injury up to HCC onset. In addition, we describe an established bioinformatics approach to highlight putative target genes and protein interaction networks where they are involved.

**Results:**

We describe four miRs (miR-125a-5p, miR-27a, miR-182, miR-193b) which showed to be differentially expressed in the chemically-induced HCC mouse model. The miRs were subjected to four of the most used predictions tools and 15 predicted target genes were identified. The expression of one (*ANK3*) among the 15 predicted targets was further validated by immunoblotting. Then, enrichment annotation analysis was performed revealing significant clusters, including some playing a role in ion transporter activity, regulation of receptor protein serine/threonine kinase signaling pathway, protein import into nucleus, regulation of intracellular protein transport, regulation of cell adhesion, growth factor binding, and regulation of TGF-beta/SMAD signaling pathway. A network construction was created and links between the selected miRs, the predicted targets as well as the possible interactions among them and other proteins were built up.

**Conclusions:**

In this study, we combined miRNA expression analysis, obtained by an *in vivo* HCC mouse model, with a bioinformatics-based workflow. New genes, pathways and protein interactions, putatively involved in HCC initiation and progression, were identified and explored.

**Electronic supplementary material:**

The online version of this article (doi:10.1186/s12859-015-0836-1) contains supplementary material, which is available to authorized users.

## Background

Hepatocellular carcinoma (HCC) is a highly aggressive epithelial tumor originating both from mature hepatocytes and stem cells [1]. It is characterized by poor prognosis and very high rate of recurrence. Epidemiological studies indicate that HCC is the fifth most common cancer and the third most common cause of cancer-related death worldwide [2]. Major risk factors include HBV/HCV infection, alcohol/drug abuse, aflatoxin exposure and genetic defects such as primary hemochromatosis and Wilson’s disease [3]. The use of animal models helped to better understand the different phases of the entire cancerous process. In this regard, animals’ treatment with diethylnitrosamine (DEN) is one of the most frequently used approaches [4]. Diethylnitrosamine is a well-known hepatic carcinogen. At the cellular level, particularly inside the hepatocyte, it acts as an alkylating agent, producing lesions and DNA mutations [5]. A study demonstrated that DEN administration for several weeks induces a rapid cancer development and promotes HCC formation in 100 % of male and 10–30 % of female mice [6]. Literature reports indicate that tumor molecular profile of mice exposed to DEN are comparable to those related to human HCC cases characterized by a poor prognosis [7]. This is the reason why it is considered a typical approach for hepatocarcinogenesis *in vivo* studies. MicroRNAs (miRNAs) are a class of small, non-coding RNAs that generated a great impact in the molecular biology field. They can negatively regulate the expression of target genes in a post-transcriptional manner, inducing mRNA degradation or inhibiting mRNA translation [8]. After their discovery, miRNAs received enormous attention because of their ability to regulate almost every aspect of cellular functions, such as differentiation, development, apoptosis and proliferation [9]. MiRNA deregulated activity has been described in various pathologies including cancer [10]. In order to make easier the identification of specific target genes, bioinformatics tools have been set-up. They provide the possibility to analyze a particular sequence located at the 5’ end of miRNA, called “seed region”, in order to predict the most probable genes potentially interacting with it. Although complementarity remains the main feature, the tools take into account other important characteristics such as site accessibility, sequence conservation, multiple binding sites [11]. Bioinformatics tools have greatly improved methods for detection of miRNA targets, due to their ability in quickly processing huge datasets. Looking at literature, some reports describe the exploitation of these algorithms to make prediction about miRNAs-target gene interactions for HCC, but the majority of these studies halted to the miRNA profiling and the validation of target genes for a specific miRNA [12, 13]. In this paper, we moved forward in order to obtain a list of potential genes, all together related to a small group of significantly altered miRNAs in HCC. So, we started to predict putative target genes by making use of relatively different bioinformatics algorithms [14–18]. Secondly, we conducted enrichment annotation analysis to identify functional clusters which could be related to those target genes. Finally, we built up networks to visualize the possible circuits and pathways where the selected miRNAs could be involved, providing a resource for further functional studies on HCC pathogenesis.

## Results

### Histological analysis

**Fig. 1:**
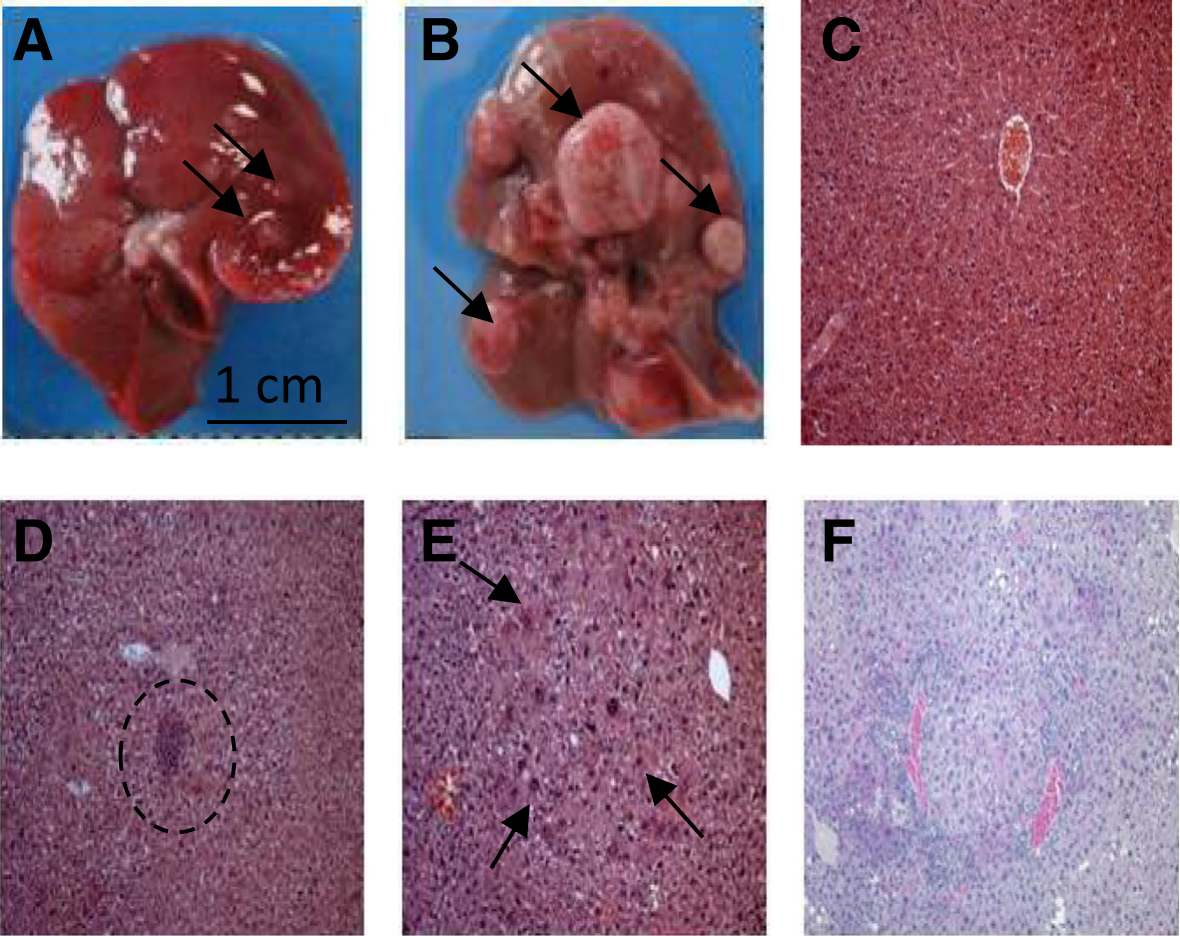
Progressive liver damage induced by DEN. **a**-**b**) Livers from mice sacrificed at 6 (**a**) and 11 months (**b**). Dark arrows indicate nodular structures visible on liver’s surface. **c**) H&E staining showing normal hepatic parenchyma (L lobe, original magnification 10X) from a control mouse sacrificed after 3 months. **d-e**) Mouse hepatic tissues (L lobe) from 3- (D, original magnification 10X) and 6-months (E, original magnification 40X) DEN-treated mice. Dashed circle and arrows indicate the presence of infiltrating lymphocytes and cellular atypia. **f**) Liver parenchyma from an 11-months DEN-treated mouse (L lobe, original magnification 10X). Marked hyperaemia, micronodules and high density of perisinusoidal lymphocytes are detected

Livers from DEN-treated and control mice were subjected to gross anatomical examination and microscopic analysis. Little nodular structures (0.1–0.2 cm maximal dimension) were observed in 20 % of mouse livers belonging to 6 months DEN-treated animals (Fig. 1a), whereas all of the mouse livers from 11 months group developed voluminous hepatic nodules (Fig. 1b). In total, fifty two nodular structures with maximal dimensions ranging from 0.3 to 2 cm were excised. Infiltrating lymphocytes were shown already after 3 months from DEN treatment, indicating the presence of inflammatory processes (Fig. 1d). In particular, 50 % of hepatic tissue samples from 3 months DEN-treated animals showed infiltrating lymphocytes, and this percentage increased up to 70 % in the 6 months DEN-treated group. Regarding the 11 months group, it was observed that 100 % of samples were characterized by lymphocyte infiltration (Fig. 1f). Histological evaluation showed typical dysplastic alterations in samples from 6 months group (Fig. 1e). Moreover, 11 months-hepatic tissues exhibited particular histological features such as hyperaemia, neo-angiogenesis, micronodules and wide fibrotic branches, showing a specific feature disrupting the normal hepatic lobular architecture (Fig. 1f).

### MiRNAs expression

Pooled RNAs from whole hepatic tissues of mice sacrificed after 3, 6 and 11 months were subjected to miRNAs’ expression analysis. Several miRNAs were found differentially expressed in hepatic tissues from DEN-treated animals, with respect to those from control mice, after 3, 6, and 11 months, and in tumors with respect to controls or peritumor liver tissues from DEN-treated animals. Among them, and after a literature review, we focused our analysis on four miRNAs (miR-27a, miR-125a-5p, miR-182, miR-193b), whose dysregulated expression was already described in hepatocarcinogenesis [19–26] and also observed in a high-fat diet-induced hepatocarcinogenesis C57BL/6J model we are analyzing (Tessitore et al., “unpublished observations”), further supporting their putative role in liver cancer. We monitored the miRNA expression level during the above-mentioned time-points (Table 1). An expression increase of miR-125a-5p, which was up-regulated after 11 months, was detected in DEN mice. MiR-182 was iso-expressed in DEN-treated groups at 3 and 6 months, whereas it was found to be up-regulated at 11 months. MiR-193b appeared iso-expressed or weakly over-expressed during the experimental time points in DEN-treated animals. MiR-27a appeared to be down-regulated at 3 months and showed high expression level at 6 and 11 months in DEN-treated mice with respect to controls. MiR-125a-5p, miR-182, miR-193b and miR-27a resulted all over-expressed in tumors in comparison to hepatic tissues from control mice, whereas miRNA-125a-5p, miR-182 and miR-193b displayed over-expression or slight increase in the comparison between tumors and paired hepatic tissues from DEN-treated mice.

**Table 1:** Relative expression of miRNAs considered for the analysis in livers and tumors

	RQ 3 M	RQ 6 M	RQ 11 M	RQ 11 M	RQ 11 M
	(DEN/CTR)	(DEN/CTR)	(DEN/CTR)	(DEN-T/CTR)	(DEN-T/DEN)
125 a-5p	0,75	0,92	2,2	3,34	1,52
182	0,97	1,05	3,52	10,09	2,86
193 b	1	1,65	1,33	4,89	3,67
27 a	0,17	15,72	4,27	4,89	1,11

RQ value is the relative quantification of miRNA expression, depending on the treatment’s length (3, 6, 11 months), obtained by comparing hepatic samples from DEN-treated (DEN), controls (CTR), and tumor samples (DEN-T) from DEN-treated mice, as indicated in the round brackets. Results are mean of 3 iterations

### Target prediction

**Fig. 2:**
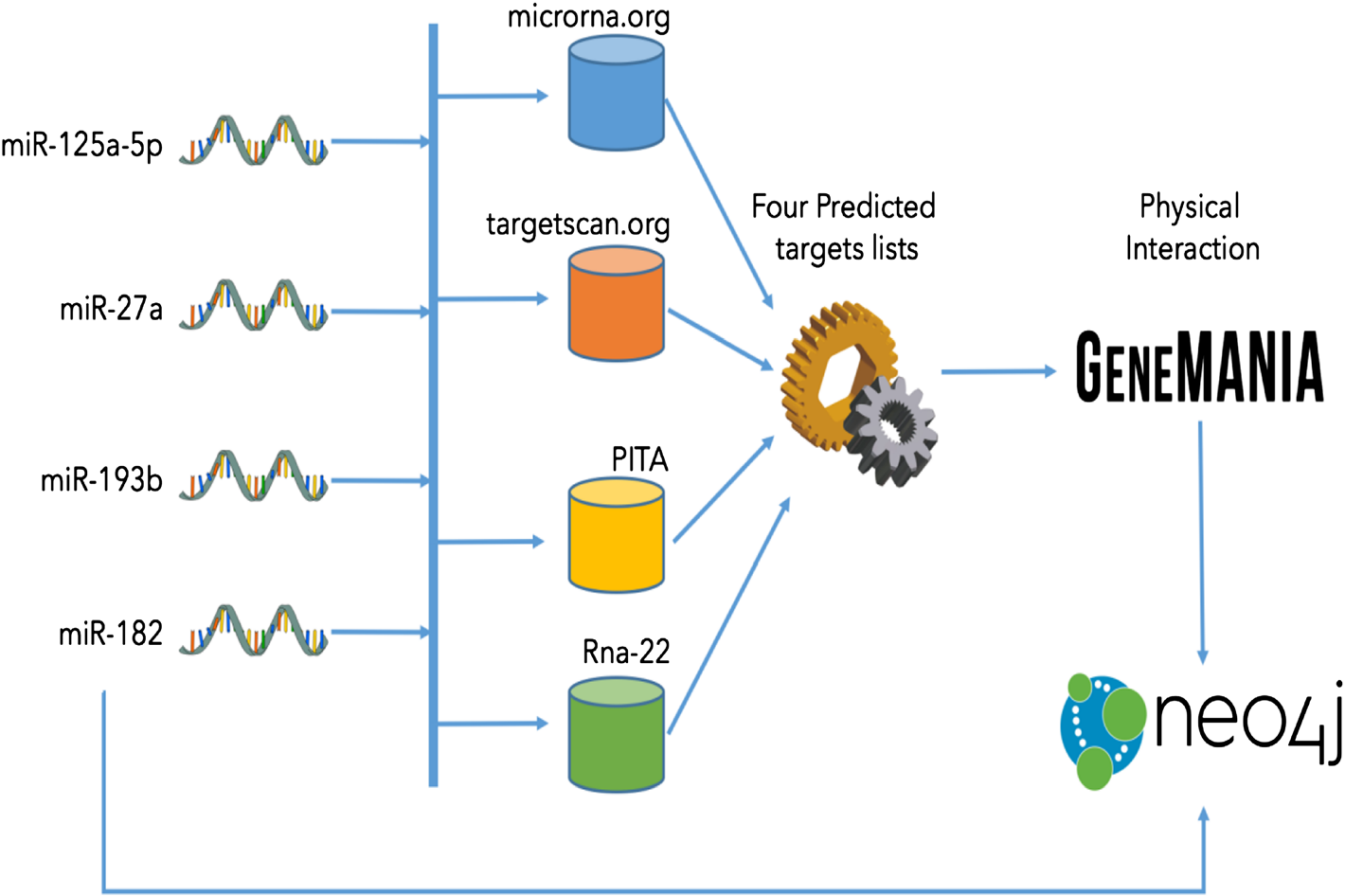
Workflow of the miRNA analysis. MiR-125a-5p, miR-27a, miR-193b and miR-182 have been considered in our analysis. We look for their common targets on four on-line DB (microrna.org, Targetscan.org, PITA and Rna-22) in order to obtain four predicted targets lists, one for each DB. From these lists, only 15 targets have been considered. We gave the filtered list of targets as input to GENEMANIA in order to obtain a physical relation network. Finally, we built up a network showing the relationships between miRNAs and targets, as well as those among targets by using Neo4j

**Fig. 3:**
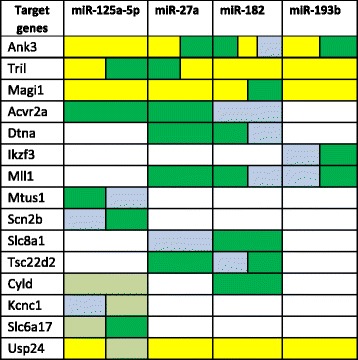
Schematic diagram illustrating the resulting 15 potential top targets for the selected microRNAs. The list includes only genes predicted by at least 2 of 4 prediction tools. Blank boxes represent too low (under the considered cut-off, see “Enrichment annotation analysis and network construction” section in “Materials and Methods”) or null association with microRNAs. Genes predicted by miRanda  genes predicted by TargetScan  genes predicted by PITA  genes predicted by Rna-22

A bioinformatics workflow focused on identifying putative target genes with respect to our selected microRNAs was set up and used for the study. A schematic representation is provided in Fig. 2. The first step of analysis involved the construction of a local database (DB) that contained information related to miRNAs and target prediction for *Mus musculus* (Table 2). The selected microRNAs were analyzed by MiRanda, TargetScan, PITA, and Rna-22, which are some of the most used target prediction tools, in order to identify target genes all at once regulated by the four microRNAs. The first two programs make predictions employing a conservation filter, whereas PITA and Rna-22 are based on free-energy criteria. MiRanda generated two lists including all targets with a good miRSVR score and a good conservation score, respectively. Then, it searched for links among the lists and created an intersecting final table comprising all target genes with the highest two types of scores. In order to work with stringent criteria and to limit false positive prediction results, a list of 29 “Good miRSVR score, non-conserved miRNA” genes, containing the most probable target genes potentially interacting with those miRNAs, was obtained. Selected microRNAs were then subjected to TargetScan, by taking into consideration the Pct score for measuring site conservation and for better translating the results from mouse to human. From the “Conserved Family Info Result” table, a total number of 148 genes was obtained. PITA and Rna-22 analysis resulted in a final group of 91 and 178 genes, respectively. All the data are described in more detail in an additional file (see Additional file 1: Table S1). For the final analysis, we decided to consider only genes predicted by at least 2 of the above-mentioned 4 prediction programs. *ANK3* mRNA was the unique target predicted by three different programs (MiRanda, TargetScan, PITA). In addition to *ANK3*, fourteen mRNAs were predicted by 2 different programs (Fig. 3, Additional file 2: Table S2).

**Table 2:** Databases and Tables used for miRNAs/targets interaction analysis

DB name	Considered table
Mirbase Data Base	mirnaTable
Microrna Data Base	gcpredSCTable
PITA Data Base	PITA_sites_mm9_0_0_ALL table
RNA22 Data Base	mus_musculus_ensembl 65 table
TargetScan Mouse Data Base	Conserved Family Info table

The on-line DB (left) and the respective specific tables considered to extract targets’ information (right) are reported

#### ANKG expression in livers and tumors

**Fig. 4:**
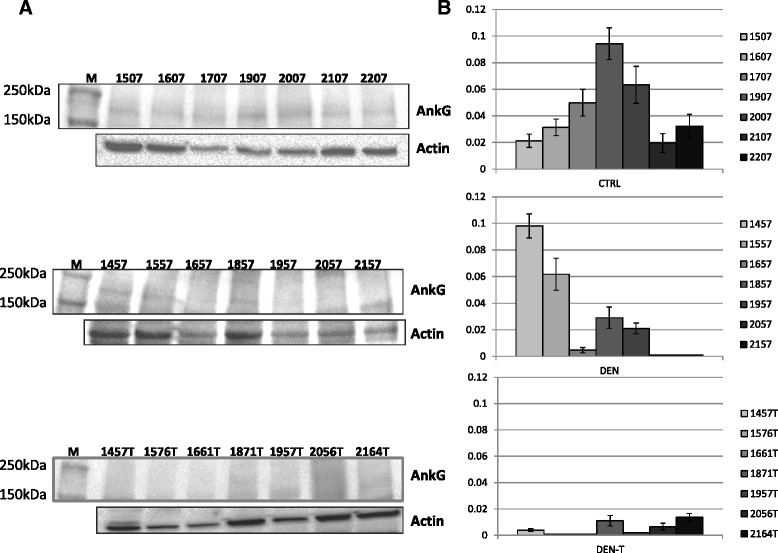
Ankyrin-G expression levels in liver tissues and tumors. **a** From the top to the bottom: AnkG expression in liver tissues from 11 months old untreated control mice, DEN-treated mice, and tumors, each excised from the corresponding mouse analyzed in the previous DEN panel (i.e., 1457-1457T, 1557-1576T, etc.). Numbers indicate mice ID, M marker. **b** Densitometric analysis of the western blots presented in A. The intensity of the bands of AnkG was normalised to the intensity of the actin bands, and the obtained values were plotted (Y axis). The actin reference value was arbitrarily set to 1

In order to assess the applicability and pertinence of the described workflow, liver tissues from 11 months DEN-treated and control mice as well as tumors from 11 months DEN-treated mice were analyzed for the expression of ANKG, which is the protein product of *ANK3*. ANKG isoforms at 200, 170, 120, and 105 kDa have been detected in mouse tissues [27]. Results from immunoblotting (Fig. 4) show a weaker expression of the 170 kDa ANKG isoform in liver tissues from 11 months DEN-treated mice with respect to those from 11 months controls, and a further reduced ANKG expression is observed in tumors from 11 months DEN-treated animals. The results are in line with those evidenced in miRNA expression analysis, which, on the contrary, show a corresponding miRNAs’ expression level increase in DEN tissues and tumors. The evidences obtained provide a validation of *in silico* data.

#### Enrichment annotation analysis and network construction

**Fig. 5:**
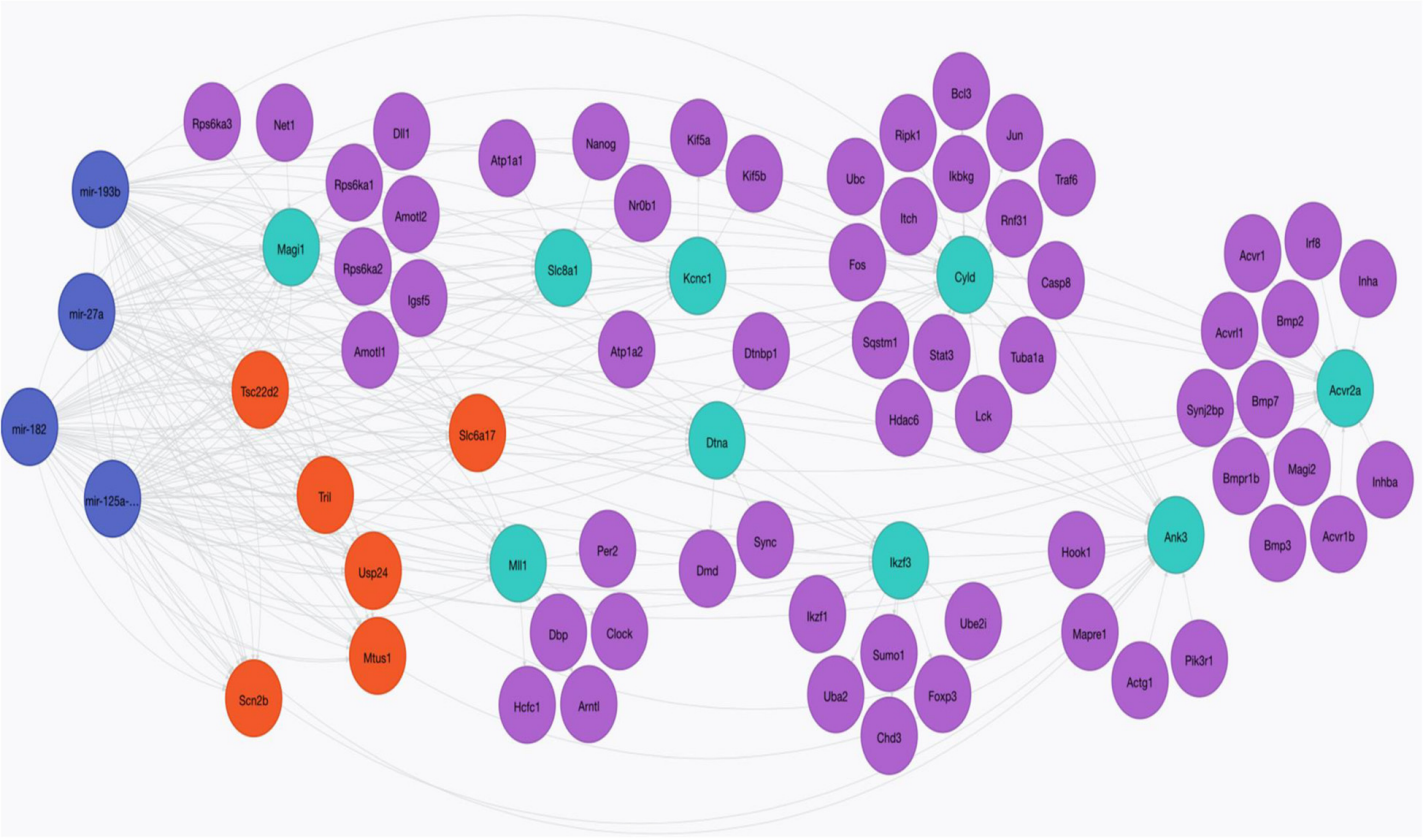
Neo4J software graphical visualization of miRNA-target interactions concerning the four considered miRNAs in HCC. Blue circles represent miRNAs. Green circles represent top target genes that are physically linked with secondary genes indicated by purple circles. Orange circles represent top target genes that have no physical interactions. Results are from Genemania elaboration. Edges indicate physical interactions

For the network construction and the enrichment annotation step, we took advantage of Genemania [28], which operated a schematic clusterization of the gene list, also reporting FDR (False Discovery Rate) and gene coverage for each cluster. The results obtained were included in a Neo4J database [29] to provide a graphical representation of miRNAs-targets interactions, including the 15 top target genes as well as 59 secondary genes (Fig. 5). Concerning the 15 top target genes, we extracted 26 significant clusters (FDR ≤ 0.05) regarding multiple cellular mechanisms such as ion transporter activity, regulation of receptor protein serine/threonine kinase signaling pathway, protein import into nucleus, regulation of intracellular protein transport, regulation of cell adhesion, growth factor binding, positive regulation of pathway-restricted SMAD protein phosphorylation (see Additional file 3: Table S3). Five genes (*SCN2B, SLC8A1, KCNC1, ACVR2A, CYLD*) appear with a “5-times” average in those categories. Moreover, in order to check for significant categories specifically related to hepatic cancer, we extended the analysis to the whole 59 secondary genes set and we obtained 20 significant clusters (Additional file 4: Table S4). In addition, we focused the analysis on a subset of 33 out of 59 secondary genes which, on the basis of PubMed search results, appeared to be specifically described in liver tumor pathogenesis (Additional file 5: Table S5). In this case, we collected 14 significant functional clusters (Additional file 6: Table S6). Regulation of TGF-beta/SMAD signaling pathway, which is known to be involved in liver damage and HCC [30], was evidenced among the most significant and important clusters identified by the above-mentioned data elaborations (Additional file 5: Table S5).

## Discussions

In the present study we show the results of a massive analysis for retrieving possible targets and pathways involved in the initiation and development of chemically-induced hepatocellular carcinoma, by using the *C57BL/6J* mouse model. The adoption of a combination of microRNAs analysis and computational-based approach allowed us to produce a set of 15 top genes predicted to be the best potential targets for four microRNAs (miR-125a-5p, miR-182, miR-193b and miR-27a). The following enrichment annotation analysis, performed both on the 15 top genes and the resulting secondary genes, allowed us to identify, among several pathways and genes involved in, some which plays an important role in liver tumor pathogenesis (i.e., TGF-beta/SMAD signaling pathway). Among the 15 top targets, some protein products have been already described in oncogenesis and metastasis. *ANK3* seems to be the most solid candidate, being predicted by 3 out of 4 programs: in particular, it results a suitable target for miR-182, which is greatly up-regulated at 11 months in tumor samples compared to controls. *ANK3,* in mouse, is distributed in several cell types, especially in renal, hepatic, muscular and nervous tissues, where different isoforms (200, 170, 120, and 105 kDa) have been detected [27]. Our results show expression decrease of the protein product ankyrin G (170 kDa) in liver tissues from DEN-treated with respect to those from control mice, and a further reduced expression in tumors with respect to DEN peritumor tissues. Data are in line with the contextual expression levels of miRNAs in hepatic tissues and tumors, providing evidence about the effectiveness of the procedure here presented and used. ANK3, member of the ankyrin protein family (*ANK1, ANK2* and *ANK3*), is typically known as epithelial ankyrin. Its protein product acts as a bridge between the plasma membrane and cytoskeleton, where it links spectrin-proteins to integral membrane proteins and is involved in regulating cellular functions such as cell motility and proliferation [31]. In several human cancer types, *ANK3* appeared to be down-regulated contributing to a poor prognosis [32]. Some authors proposed a possible connection between *ANK3* dysregulation and epithelial-to-mesenchymal transition (EMT) [33]. They stated that decreased levels of Ankyrin-G in tumoral cells caused by the EMT process could free-up the neurotrophin receptor-interacting melanoma antigen (NRAGE) for translocation into the nucleus, where it could interact with the repressor protein TBX2 to suppress *p14ARF* expression. In normal cells, p14ARF acts to block NRAGE-TBX2 complex, allowing NRAGE sequestration by Ankyrin-G. This confers sensitivity to “anoikis”, a specific apoptosis program activated when cells are able to detach from extracellular matrix and adhere to other substrates in order to limit their migration potential. Therefore, it could be hypothesized that *p14ARF* down-regulation could protect cancer cells from anoikis activation. An indirect confirmation of the *ANK3*/EMT relation comes from an interesting work [34] where the role of HOOK1, an interaction ANK3 partner we previously observed in Genemania network (see “Enrichment annotation analysis and network construction” section in “Materials and Methods”), was described in this phenomenon. The authors showed that HOOK1 displayed a regulatory effect on EMT, since its overexpression led to EMT inhibition. Conversely, reduced HOOK1 expression level contributed to EMT phenomenon. The existence of a physical link for Ankyrin G and HOOK1 provide thus an additional clue to the hypothesized role of ANK3 in the regulation of EMT for tumor metastases [35]. Another compelling mechanism involving a possible role of *ANK3* in cancer pathogenesis is presented by an elegant work by Ignatiuk et al. [36] where it was demonstrated that smaller isoforms of ANK3, called ANK120 and ANK105, were able to bind the PDGFR-binding subunit of PI3K, p85, influencing the lysosomal degradation of the receptor. These proteins, particularly expressed in liver, lack the repeat domain which is responsible for their positioning on plasma membrane and for this reason they localize to late endosomes and lysosomes to target materials to be degraded [37]. ANK120 and-105 overexpressing-cells exhibited a rapid degradation followed by reduced levels of PDGF receptor and an overall minor sensitivity to proliferation upon PDGF stimulation. In HCC cells, down-regulation of these isoforms could lead to delayed PDGFR degradation that could give rise to a sustained signalling of PDGF and downstream pathways, resulting in enhanced proliferation. Keeping in mind that *PDGFR* expression is reported to be up-regulated in HCC human livers [38], focused analysis of this mechanism could be very intriguing to explore.

CYLD, here identified as a potential miRNAs target and in functional clusterization, is a protein implicated in the regulation of protein localization and transport inside the cells. *CYLD* is a gene whose loss or mutations predispose to the development of human cylindroma, a particular type of hair follicle benign tumor. It encodes for a deubiquitinating enzyme, ubiquitously expressed, which is depicted as a tumor suppressor since it lacks its expression in various human tumor types [39]. CYLD removes ubiquitin chains from different molecules, such as TNF receptor-associated factor 2/6 (TRAF2/6) and B-cell limphoma-3 (BCL-3), thus participating in pathways involved in cell proliferation and survival [40]. Literature reports illustrated some mechanisms specifically involving this gene in tumor tissues. Pannem et al. [41] showed that *CYLD*-deficent mice developed hepatic tumors after DEN-treatment, using an animal model similar to that here described. At the molecular level, *CYLD* down-regulation led to robust activation of *JNK1* which reflected in *c-Myc* levels stabilization and enhanced transcription of *cyclin-D1*. This mechanism could justify the elevated proliferation rates observed in HCC cells. On the other hand, other researchers demonstrated also direct CYLD cross-talk with NF-kB pathway [42]. In this case, CYLD influenced this pathway through the control of BCL-3 localization. Indeed, BCL-3 was able to associate with NF-kB heterodimers and triggered the transcription of NF-kB-related genes such as *cyclin-D1*, but only when it was inside the nucleus [43]. In normal cells, BCL-3 was retained into the cytoplasm, due to the elimination of Lys-63 polyubiquitin chains, a task mediated by CYLD itself. During transformation, growth-promoting stimuli produced BCL-3 transfer from the cytoplasm to the perinuclear region. Here, decreasing *CYLD* levels activated BCL3 accumulation followed by its import into the nucleus, probably mediated by an interaction between the polyubiquitin chains of BCL-3 and importins. Once inside the nucleus, BCL-3 can bind to NF-kB to form a complex that was able to selectively recognize NF-kB binding sites, stimulating trascription of oncogenes. Based on our findings, *CYLD* was predicted to be a target of miR-125a-5p and miR-182. Effectively, *CYLD* is validated for human miR-182, according to miRTarBase (ISBLab) [44]. In light of all these evidences, we can deduce that miR-125a-5p and miR-182 could modify *CYLD* expression in cancerous tissues causing a marked reduction.

Clusterization analysis reveals the presence of *SLC8A1* as an additional gene targeted by the selected microRNAs. Our data showed that this gene could be regulated by miR-182 and miR-27a which have been already described as over-expressed microRNAs in human HCC cells [20, 24, 25]. *SLC8A1* belongs to the large family of solute carrier (SLC) transporters, proteins designated to transport different molecules including nutrients, metals, ions and drugs across the membranes [45]. Generally, they are influx transporters and *SLC8A1* represents one of them. This gene encodes a protein, called NCX1, which regulates the extra- and intracellular levels of sodium (Na^+^) and calcium (Ca^++^), allowing Na^+^ influx and Ca^++^ efflux in normal conditions [46]. NCX1, known as an important Ca^++^ exchanger in plasma membrane, could invert this flux in pathological conditions, determining Ca^++^ influx. This situation provokes a rise of intracellular calcium levels and the consequent activation of Ca^++^-dependent signaling pathways that stimulate specific responses, such as apoptosis activation. In some human cancers, *SLC8A1* expression was shown to be decreased, producing a corresponding reduction of intracellular calcium levels which leads to apoptosis evasion and more sustained proliferation rate [47]. Moreover, as described by Januchowski et al. [48], *SLC8A1* was addressed as responsible for multidrug resistance, one of the big problems in chemotherapeutic treatments. In this context, the authors documented a strong *SLC8A1* downregulation in several drug-resistant ovarian cancer cell lines, claiming that it could represent an interesting therapeutic target for this malignancy. These data fit with our results; indeed, miR-27a and miR-182 up-regulation might be responsible of *SLC8A1* ipo-expression in HCC tissues. Although there are no evidences in literature about *SLC8A1* expression level in HCC, a number of studies have described its lowered activity in other human cancers [47, 48]. Hence, it could be useful to evaluate its expression also in another completely different environment, like HCC could be.

Another interesting gene linked to our group of selected miRNAs and comprised in the 15 top target gene set is MAGI1 (membrane-associated guanylate kinase, WW and PDZ domain containing 1), which is a scaffold protein whose principal activity is to regulate and stabilize cell-cell contacts [49]. Because of this activity, MAGI1 is considered an important regulator for cell junctions with tumor suppressor behaviour. Some studies elucidated its functions stating that it recruits PTEN to the E-cadherin complexes located at junctional sites and, subsequently, induce PTEN to downregulate phosphatidylinositol-3,4,5 trisphosphate pools, leading to the activation of the effector downstream molecules [50]. Zhang et al. reported MAGI1 downregulation at mRNA and protein level in HCC human tissues and they found a direct correlation between its reduced expression and a poor prognosis [51]. These results fitted with our predictions. In fact, our data indicated MAGI1 as a putative target for all of the four miRNAs, in particular for miR-182. Considering that all of these miRNAs were up-regulated in tumor tissues in our system, we could speculate that one or more of them could block the expression of MAGI1 in HCC tissues.

## Conclusions

Since their discovery, many advances were made about the understanding of microRNA functions and their possible use for biomedical purposes. Especially for cancer, an increasing number of studies highlighted their potential in defining diagnosis, prognosis and therapy and great efforts are being made to support the use of these molecules in clinical applications. In this work we combined biomolecular results, based on an *in vivo* model, with *in silico* analysis. We created a workflow able to connect miRNAs, found to be dysregulated in chemically-induced hepatocarcinogenesis, to respective putative targets; then we generated a wide protein interaction network involving other proteins physically interacting with them. The workflow was validated by evaluating and confirming the expression levels of one among the microRNAs’ predicted targets (ANKG) in the animal model here described. In conclusion, the experimental procedure could be used and employed for further researches on HCC initiation, development and progression.

## Methods

### Animal husbandry

Three groups, each composed by eight 14 days old *C57BL/6J* mice (Charles River laboratories, France) were treated with intra-peritoneal injection of diethylnitrosamine DEN (25 mg/kg) in saline solution, and sacrificed by CO_2_ asphyxiation after 3, 6, and 11 months. As many groups of as many 14 days old mice were treated, in parallel, with intra-peritoneal physiological saline solution injection, and sacrificed at the same experimental time points. Animals were fed with a standard diet (Harlan 2918, Teklad Global 18 % Protein Rodent Diet) and housed under the same conditions (T = 20-21 °C, 12 h light–dark cycle). All experimental procedures were performed in conformity with national and international laws and policies (European Economic Community Council Directive 86/609, Italian Legislative Decree 116/92, National Institutes of Health Guide for the Care and Use of Laboratory Animals) and were approved by the Internal Committee and the Italian Ministry of Health.

### Tissue collection

After sacrifice, livers were explanted and weighed. Liver tissues were stored in RNA Later (Ambion), frozen at –80 °C or fixed in formalin for further analyses.

### Histological analysis

Immediately after removal, hepatic tissue sections were fixed in 10 % neutral buffered formalin. (formaldehyde 4 % w/m, phosphate buffer 0,05M, Bio Optica). After 48 h, tissue sections were paraffin-embedded using standard procedures. Finally, 3 μm sections were stained with hematoxylin and eosin and then observed by light microscope (Nikon Eclipse E200) to visualize the general hepatic architecture.

### RNA extraction

Total RNA was extracted by using MiRVana kit (Ambion) which allows the recovery of total RNA, including the fraction less than 200 nt in lenght, according to the manufacturer’s instructions. RNA quantification and purity was measured with Nanodrop 1000 v3.7 (Thermo Scientific) and RNA quality was verified by ethidium bromide staining, after agarose gel electrophoresis.

### MiRNA analysis

Same amounts of total RNA, extracted by livers from eight mice belonging to every experimental group, were mixed together to obtain pools from DEN-treated and control mice. RNAs were also extracted from 7 tumors from 11 months DEN-treated mice (1 tumor per mouse, tumors from the eighth mouse were too small to be excised -2 mm maximal dimension-), and pooled together as well. For the analysis, 700 ng of mixed RNAs were subjected to retro-transcription by using the TaqMan MicroRNA RT kit and the Megaplex Rodent Primer Pool A (Life Technologies), and analyzed for microRNAs’ expression levels by using the TaqMan rodent MicroRNA array set A, v3.0 (Life Technologies), according to the manufacturer’s instructions. Mamm-U6 was used as endogenous control. Each RNAs’ pool was analyzed in triplicate. To collect data, ViiA-7 system and software (Life Technologies) was used, and the ΔΔCt comparative method was applied. We considered Relative Quantification values RQ ≥ 2 or RQ ≤ 0.5 as up- and down-regulation cut-off, respectively.

#### Western blot

Tissues were homogenized by using Tissue Lyser (Qiagen), and proteins were extracted in RIPA buffer. Proteins from livers and tumor tissues were recovered from the same samples used for miRNA analysis. We analyzed, at the individual level, seven samples from 11 months controls, DEN-treated mice, and tumors from paired DEN-treated mice. Thirty micrograms of total extracts were loaded onto a 4–15 % SDS-PAGE precast get (Bio-Rad). After electroblotting (250 mA, 1.5 h), proteins were stained with Ponceau, and membrane was washed, blocked with 5 % non-fat milk in TBST 1X for 1 h, and then incubated overnight at 4 °C with 1:500 anti-ANKG antibody (Santa-Cruz) in TBST 1X. The membrane was washed and incubated with the secondary anti-rabbit antibody (Santa Cruz) for 1.5 h at room temperature. After washing, SuperSignal West Pico Chemiluminescent Substrate (Thermo Scientific) was added and images were collected by using CGel Doc XR+ (Bio Rad) instrument, by using the same exposure time. Densitometric analysis was performed by using Image Lab 4.0 (Bio-Rad). Actin (Santa Cruz) was used as endogenous control, by following the same experimental conditions.

#### Target prediction analysis

Four miRNAs (miR-125a-5p, miR-182, miR-193b and miR-27a) were selected and subjected to target prediction. MySQL technology [52] was used to construct a local database. In particular, Mirbase [53] has been considered to extract data about microRNA, whereas four on-line DB were used to perform predictive analysis: MiRanda (August 2010 release) [14], TargetScan Mouse v6.2 (June 2012 release) [16–54], PITA (Segal lab of computational biology) [17], and Rna-22 (Thomas Jefferson University) [18].

According to the MiRanda authors’ discussion in [15], –1.2 was chosen as cut-off mirSVR score to obtain a reasonable empirical probability of target mRNA downregulation. Data were downloaded from the MiRanda database MirBase. For TargetScan, only genes with a probability of conserved targeting (Pct) > 0.1 were considered. Data from PITA were recovered downloading the “3/15 flank All” table in “Mouse” column in “PITA Targets catalog” and setting 10 kcal/mol as a general cutoff value. Regarding Rna-22, only genes with a binding energy less than -25 kcal/mol were included in the subsequent steps.

#### Network construction and enrichment annotation analysis

A list constituted by primary target genes was subjected to software analysis as a query list using the software Genemania v3.1.2 and then an interaction network was retrieved, selecting molecular functions as weighing method (dysplaying n.100 related genes and n.20 related attributes in the “number of gene results” field). Similar lists were built by considering also the set of 59 secondary genes and a subset of 33 genes which, on the basis of a PubMed search, appeared already described in liver cancer. Due to the higher number of input genes, the Number Gene parameters were, in this case, reduced to 10 related attributes and 10 related genes. Gene targets network construction was made using the same software Genemania v3.1.2. In particular, physical interactions between targets were considered. The Genemania “Functions” panel that allows the user to visualize all the clusters for Gene Ontology (GO) terms related to the query gene list was used. Thus, this possibility was exploited to carry out enrichment annotation analyses for our target genes list. The results hitherto obtained were included in a Neo4J database to provide a graphical representation of miRNAs-targets interactions (Fig. 5).

## Additional files

Additional file 1: Table S1. List of target genes obtained from TargetScan Mouse v 6.2, List of potential targets from PITA, List of target from Rna-22, List of target genes predicted by MiRanda. Four Excel sheets showing the four lists of potential targets for the miR 125a-5p, miR-193b, miR-182 and miR-27a obtained from the prediction softwares TargetScan Mouse, PITA, Rna-22 and MiRanda. (XLSX 32 kb) Additional file 2: Table S2. The group of fifteen top target genes inferred from target prediction analysis. A table comprising the fifteen top target genes for the miR 125a-5p, miR-193b, miR-182 and miR-27a, retrieved from at least 50 % of prediction softwares used. (DOCX 11 kb) Additional file 3: Table S3. Most significant functional groups obtained from clusterization process by Genemania (FDR ≤ 0.05). Excel table presenting the 26 most significant cluster extracted by Genemania after selection of the fifteen top target genes. The table shows the false discovery rate and the gene coverage for each cluster. (XLSX 14 kb) Additional file 4: Table S4. Most significant functional groups obtained from clusterization process by Genemania, regarding 59 secondary genes (FDR ≤ 0.05). Excel table presenting the 20 most significant cluster extracted by Genemania on the whole set of 59 secondary genes. The table shows the false discovery rate and the gene coverage for each cluster. (XLSX 11 kb) Additional file 5: Table S5. List of secondary genes from Genemania involved in liver cancer on the basis of literature (PubMed) review. Excel table presenting 33 out of 59 secondary genes, already described in liver tumor pathogenesis. (XLSX 9 kb) Additional file 6: Table S6. Most significant functional groups obtained from clusterization process by Genemania, regarding 33 secondary genes, most closely related to liver cancer (FDR ≤0.05). Excel table presenting the 14 most significant cluster extracted by Genemania after selection of the 33 secondary genes. The table shows the false discovery rate and the gene coverage for each cluster. (XLSX 11 kb)

## Abbreviations

HCC: hepatocellular carcinoma
miRNAs/miRs: microRNAs
DEN: diethylnitrosamine
DB: database
FDR: false discovery rate
EMT: epithelial-to-mesenchymal transition
NRAGE: neurotrophin receptor-interacting melanoma antigen
BCL-3: B-cell limphoma-3
Pct: probability of conserved targeting
GO: gene ontology

## References

[1] Vann Malestein H, Van Pelt J, Verslype C (2011). Molecular classification of hepatocellular carcinoma 2011. Eur J Cancer.

[2] Bosetti C, Turati F, La Vecchia C (2014). Hepatocellular carcinoma epidemiology. Best Pract Res Clin Gastroenterol.

[3] Hill-Baskin AE, Markiewski MM, Buchner DA, Shao H, De Santis D, Hsiao G (2009). Diet-induced hepatocellular carcinoma in genetically predisposed mice. Hum Mol Gen.

[4] Fausto N, Campbell JS (2010). Mouse models of hepatocellular carcinoma. Semin Liver Dis.

[5] Verna L, Whysner J, Williams GM (1996). N-nitrosodiethylamine mechanistic data and risk assessment: bioactivation, DNA-adduct formation, mutagenicity, and tumor initiation. PharmacolTher.

[6] Nakatani T, Roy G, Fujimoto N, Asahara T, Ito A (2001). Sex hormone dependency of diethylnitrosamine-induced liver tumors in mice and chemoprevention by leuprorelin. Jpn J Cancer Res.

[7] Lee JS, Chu IS, Mikaelyan A, Calvisi DF, Heo J, Reddy JK (2004). Application of comparative functional genomics to identify best-fit mouse models to study human cancer. Nat Genet.

[8] Carrington JC, Ambros V (2003). Role of microRNAs in plant and animal development. Science.

[9] Ambros V (2004). The functions of animal microRNAs. Nature.

[10] Gong H, Liu CM, Liu DP, Liang CC (2005). The role of small RNAs in human diseases: potential troublemaker and therapeutic tool. Med Res Rev.

[11] Saito T, Saetrom P (2010). MicroRNAs – targeting and target prediction. N Biotecnol.

[12] Bae HJ, Jung KH, Eun JW, Shen Q, Kim HS, Park SJ (2015). MicroRNA-221 governs tumor suppressor HDAC6 to potentiate malignant progression of liver cancer. J Hepatol.

[13] Chang RM, Yang H, Fang F, Xu JF, Yang LY (2014). MicroRNA-331-3p promotes proliferation and metastasis of hepatocellular carcinoma by targeting PH domain and leucine-rich repeat protein phosphatase. Hepatology.

[14] Betel D, Wilson M, Gabow A, Marks DS, Sander C (2008). The microRNA.org resource: targets and expression. Nucleic Acids Res.

[15] Betel D, Koppal A, Agius P, Sander C, Leslie C (2010). Comprehensive modeling of microRNA targets predicts functional non-conserved and non-canonical sites. Genome Biol.

[16] Friedman RC, Farh KK, Burge CB, Bartel DP (2009). Most mammalian mRNAs are conserved targets of microRNAs. Genome Res.

[17] Kertesz M, Iovino N, Unnerstall U, Gaul U, Segal E (2007). The role of site accessibility in microRNA target recognition. Nat Genet.

[18] Miranda KC, Huynh T, Tay Y, Ang YS, Tam WL, Thomson AM (2006). et al.:A pattern-based method for the identification of MicroRNA binding sites and their corresponding heteroduplexes. Cell.

[19] Wu XJ, Li Y, Liu D, Zhao LD, Bai B, Xue MH (2013). MiR-27a as an oncogenic microRNA of hepatitis B virus- related hepatocellular carcinoma. Asian Pac J Cancer Prev.

[20] Huang S, He X, Ding J, Liang L, Zhao Y, Zhang Z (2008). Upregulation of miR-23a ~ 27a ~ 24 decreases transforming growth factor-beta-induced tumor-suppressive activities in human hepatocellular carcinoma cells. Int J Cancer.

[21] Braconi C, Valeri N, Gasparini P, Huang N, Taccioli C, Nuovo G (2010). Hepatitis C virus proteins modulate microRNA expression and chemosensitivity in malignant hepatocytes. Clin Cancer Res.

[22] Kim JK, Noh JH, Jung KH, Eun JW, Bae HJ, Kim MG (2013). Sirtuin7 oncogenic potential in human hepatocellular carcinoma and its regulation by the tumor suppressors MiR-125a-5p and MiR-125b. Hepatology.

[23] Wang J, Li J, Shen J, Wang C, Yang L, Zhang X (2012). MicroRNA-182 downregulates metastasis suppressor 1 and contributes to metastasis of hepatocellular carcinoma. BMC Cancer.

[24] Wang C, Ren R, Hu H, Tan C, Han M, Wang X (2014). MiR-182 is up-regulated and targeting Cebpa in hepatocellular carcinoma. Chin J Cancer Res.

[25] Wang TH, Yeh CT, Ho JY, Ng KF, Chen TC (2015). OncomiR miR-96 and miR-182 promote cell proliferation and invasion through targeting ephrinA5 in hepatocellular carcinoma. Mol Carcinog.

[26] Leung WK, He M, Chan AW, Law PT, Wong N (2015). Wnt/β-Catenin activates MiR-183/96/182 expression in hepatocellular carcinoma that promotes cell invasion. Cancer Lett.

[27] Peters LL, John KM, Lu FM, Eicher EM, Higgins A, Yialamas M (1995). Ank3 (Epithelial Ankyrin), a Widely Distributed New Member of the Ankyrin Gene Family and the Major Ankyrin in Kidney, Is Expressed in Alternatively Spliced Forms, Including Forms That Lack the Repeat Domain. J Cell Biol.

[28] Montojo J, Zuberi K, Rodriguez H, Kazi F, Wright G, Donaldson SL (2010). GeneMANIA Cytoscape plugin: fast gene function predictions on the desktop. Bioinformatics.

[29] Robinson I, Webber J, Eifrem E. Graph Databases. 2nd ed. Sebastopol, CA USA: O'Reilly Media, Inc., 2013.

[30] Dooley S, ten Dijke P (2012). TGF-β in progression of liver disease. Cell Tissue Res.

[31] Lambert S, Bennett V (1993). From anemia to cerebellar dysfunction. A review of the ankyrin gene family. Eur J Biochem.

[32] Glinski GV, Berezovska O, Glinskii AB (2005). Microarray analysis identifies a death-from cancer signature predicting therapy failure in patients with multiple types of cancer. J. Clin. Invest..

[33] Kumar S, Park SH, Cieply B, Schupp J, Killiam E, Zhang F (2011). A Pathway for the Control of Anoikis Sensitivity by E-Cadherin and Epithelial-to-Mesenchymal Transition. Mol Cell Biol.

[34] Li S, Wang L, Zhao Q, Liu Y, He L, Xu Q (2014). SHP2 positively regulates TGFβ1-induced epithelial-mesenchymal transition modulated by its novel interacting protein Hook1. J Biol Chem.

[35] Weimer JM, Chattopadhyay S, Custer AW, Pearce DA (2005). Elevation of Hook1 in a disease model of Batten disease does not affect a novel interaction between Ankyrin G and Hook1. Biochem Biophys Res Commun.

[36] Ignatiuk A, Quickfall JP, Hawrysh AD, Chamberlain MD, Anderson DH (2006). The smaller isoforms of ankyrin 3 bind to the p85 subunit of phosphatidylinositol 3' kinase and enhance platelet-derived growth factor receptor down-regulation. J Biol Chem.

[37] Hoock TC, Peters LL, Lux SE (1997). Isoforms of ankyrin-3 that lack the NH2-terminal repeats associate with mouse macrophage lysosomes. J Cell Biol.

[38] Chu JS, Ge FJ, Zhang B, Wang Y, Silvestris N, Liu LJ (2013). Expression and prognostic value of VEGFR-2, PDGFR-β, and c-Met in advanced hepatocellular carcinoma. J Exp Clin Cancer Res.

[39] Kinoshita H, Okabe H, Beppu T, Chikamoto A, Hayashi H, Imai K (2013). CYLD downregulation is correlated with tumor development in patients with hepatocellular Carcinoma. Mol Clin Oncol.

[40] Massoumi R (2010). Ubiquitin chain cleavage: CYLD at work. Trends Biochem Sci.

[41] Pannem RR, Dorn C, Ahlqvist K, Bosserhoff AK, Hellerbrand C, Massoumi R (2014). CYLD controls c-MYC expression through the JNK-dependent signaling pathway in hepatocellular carcinoma. Carcinogenesis.

[42] Massoumi R, Chmielarska K, Hennecke K, Pfeifer A, Fassler R (2006). Cyld inhibits tumor cell proliferation by blocking Bcl-3-dependent NF-kappaB signaling. Cell.

[43] Park SG, Chung C, Kang H, Kim JY, Jung G (2006). Up-regulation of Cyclin D1 by HBx Is Mediated by NF-B2/BCL3 Complex through kappaB Site of Cyclin D1 Promoter. J Biol Chem.

[44] Hsu SD, Tseng YT, Shrestha S, Lin YL, Khaleel A, Chou CH (2014). miRTarBase update 2014: an information resource for experimentally validated miRNA-target interactions. Nucleic Acids Res.

[45] Hediger MA, Clemencon B, Burrier RE, Bruford EA (2013). The ABCs of membrane transporters in health and disease (SLC series): introduction. Mol Aspects Med.

[46] Khananshvili D (2013). The SLC8 gene family of sodium-calcium exchangers (NCX) - structure, function, and regulation in health and disease. Mol Aspects Med.

[47] Munoz JJ, Drigo SA, Barros-Filho MC, Marchi FA, Scapulatempo-Neto C, Pessoa GS (2014). Down-Regulation of SLC8A1 as a Putative Apoptosis Evasion Mechanism by Modulation of Calcium Levels in Penile Carcinoma. J Urol.

[48] Januchowski R, Zawierucha P, Rucinski M, Andrzejewska M, Wojtowicz K, Nowicki M (2014). Drug transporter expression profiling in chemoresistant variants of the A2780 ovarian cancer cell line. Biomed Pharmacother.

[49] Shiratsuchi T, Futamura M, Oda K, Nishimori H, Nakamura Y, Tokino T (1998). Cloning and characterization of BAI-associated protein 1: a PDZ domain-containing protein that interacts with BAI1. Biochem Biophys Res Commun.

[50] Kotelevets L, van Hengel J, Bruyneel E, Mareel M, van Roy F, Chastre E (2005). Implication of the MAGI-1b/PTEN signalosome in stabilization of adherens junctions and suppression of invasiveness. FASEB J.

[51] Zhang G, Liu T, Wang Z (2012). Downregulation of MAGI1 associates with poor prognosis of hepatocellular carcinoma. J Invest Surg.

[52] Widenius M, Axmark D. Mysql Reference Manual. 1st ed. Paul DuBois. Sebastopol, CA USA: O'Reilly & Associates Inc., 2002.

[53] Griffiths-Jones S, Grocock RJ, Van Dongen S, Bateman A, Enright AJ (2006). miRBase: microRNA sequences, targets and gene nomenclature. Nucleic Acids Res.

[54] Lewis BP, Burge CB, Bartel DP (2005). Conserved seed pairing, often flanked by adenosines, indicates that thousands of human genes are microRNA targets. Cell.

