# Supplementary material for: Bioinformatics approach to predict target genes for dysregulated microRNAs in hepatocellular carcinoma: study on a chemically-induced HCC mouse model
Source: BMC Bioinformatics. 2015 Dec 10;16:408. doi: 10.1186/s12859-015-0836-1 (PMC4676132; doi:10.1186/s12859-015-0836-1)
Supplement: Additional file 2: Table S2. — The group of fifteen top target genes inferred from target prediction analysis. A table comprising the fifteen top target genes for the miR 125a-5p, miR-193b, miR-182 and miR-27a, retrieved from at least 50 % of prediction softwares used. (DOCX 11 kb) [file 12859_2015_836_MOESM2_ESM.docx]

**Table S2. The group of fifteen top target genes inferred from target prediction analysis**.

| **Gene Symbol** | **Ensemble ID** |
| --- | --- |
| Tril | ENSMUSG00000043496 |
| Magi1 | ENSMUSG00000045095 |
| Acvr2a | ENSMUSG00000052155 |
| Dtna | ENSMUSG00000024302 |
| Ikzf3 | ENSMUSG00000018168 |
| Mll1 | ENSMUSG00000002028 |
| Mtus1 | ENSMUSG00000045636 |
| Scn2b | ENSMUSG00000070304 |
| Slc8a1 | ENSMUSG00000054640 |
| Tsc22d2 | ENSMUSG00000027806 |
| Cyld | ENSMUSG00000036712 |
| Kcnc1 | ENSMUSG00000058975 |
| Slc6a17 | ENSMUSG00000027894 |
| Usp24 | ENSMUSG00000028514 |
| Ank3 | ENSMUSG00000069601 |
